# Neuronal plasticity in a case with total hemispheric lesion

**Published:** 2011-08-25

**Authors:** M Ipek, H Hilal, T Nese, M Aynur, E Gazanfer

**Affiliations:** * Marmara University Hospital, Department of NeurologyTurkey; ** Marmara University Hospital, Department of Neurology, NeuropsychologyTurkey; *** Marmara University Hospital, Department of RadiologyTurkey

**Keywords:** Neuronal plasticity, reorganization, hemispheric lesion, functional MRI

## Abstract

**Rationale**: The adult brain maintains the ability for reorganization or plasticity throughout life. Non– invasive techniques such as functional magnetic resonance imaging (f–MRI), Transcranial Magnetic Stimulation (TMS) and magnetoencephalography could be used to show recovery of function after stroke.

**Objective**: Explanation of neuronal plastisity and intra–hemisferic reorganization with functional recovery of a case who has total extensive damage on the left hemisphere.

**Case Report**: Twenty–five years old female patient was admitted to our hospital with a right–sided sequel hemiparesis and homonymous hemianopia. She had right–sided paresia after an inguinal hernia operation when she was one and a half years old. On neurological examination she was speaking fluently, and cooperated on complicated comments. Motor examination revealed right–sided spastic hemiparesis predominant on distal parts and right sided visual field defect. But she was continuing her activity of daily livings without help. Her detailed neuropsycological examination revealed mild cognitive dysfunction. Cranial MRI showed total left hemisphere encephalomalasia. Right hemisphere function was noticed on task–related brain activation during voluntary movement of her right leg (she was not capable of performing right hand function tasks by herself ) on functional MRI.

**Conclusion**: Cerebral lesions in the early life can be compensated with the unaffected hemisphere by the neuronal reorganisation and a patient with complete left hemisphere lesion such as our patient can speak, maintain her life without assistance but with mild cognitive decline, compared with elderly stroke patients.

## Introduction

Nervous system has a property to reorganize its function after a lesion or environmental change [1]. The mature stage of the CNS at the time of the insult for this reorganization is very important [2]. If a lesion occurs at the early childhood, the restoration of neurological function takes a different process from adulthood. The structural properties, location and extent of the lesion also affect the reorganization of the brain as well as the time of the insult [2].

Neuronal plasticity has been shown by many experimental studies and clinical experiences, and non-invasive techniques such as functional magnetic resonance imaging (f–MRI), transcranial magnetic stimulation (TMS) and magnetoencephalography (MEG) allow understanding of the functional adaptive changes in human beings [3–5]. Most of the studies about neuronal plasticity are related to stroke patients. It's amazing how cerebral networks respond to focal injury [3]. A lesion of the left hemisphere occurred during childhood rarely result in speech and language disorders [6].

We report a patient with mild motor and cognitive deficit despite a total left hemisphere lesion and we explain the function of neuronal reorganization in early stage of life.

Nervous system has a property to reorganize its function after a lesion or environmental change [1]. The mature stage of the CNS at the time of the insult for this reorganization is very important [2]. If a lesion occurs at the early childhood, the restoration of neurological function takes a different process from adulthood. The structural properties, location and extent of the lesion also affect the reorganization of the brain as well as the time of the insult [2].

Neuronal plasticity has been shown by many experimental studies and clinical experiences, and non–invasive techniques such as functional magnetic resonance imaging (f–MRI), transcranial magnetic stimulation (TMS) and magnetoencephalography (MEG) allow understanding of the functional adaptive changes in human beings [3–5]. Most of the studies about neuronal plasticity are related to stroke patients. It's amazing how cerebral networks respond to focal injury [3]. A lesion of the left hemisphere occurred during childhood rarely result in speech and language disorders [6].

We report a patient with mild motor and cognitive deficit despite a total left hemisphere lesion and we explain the function of neuronal reorganization in early stage of life.

**Figure 1:**
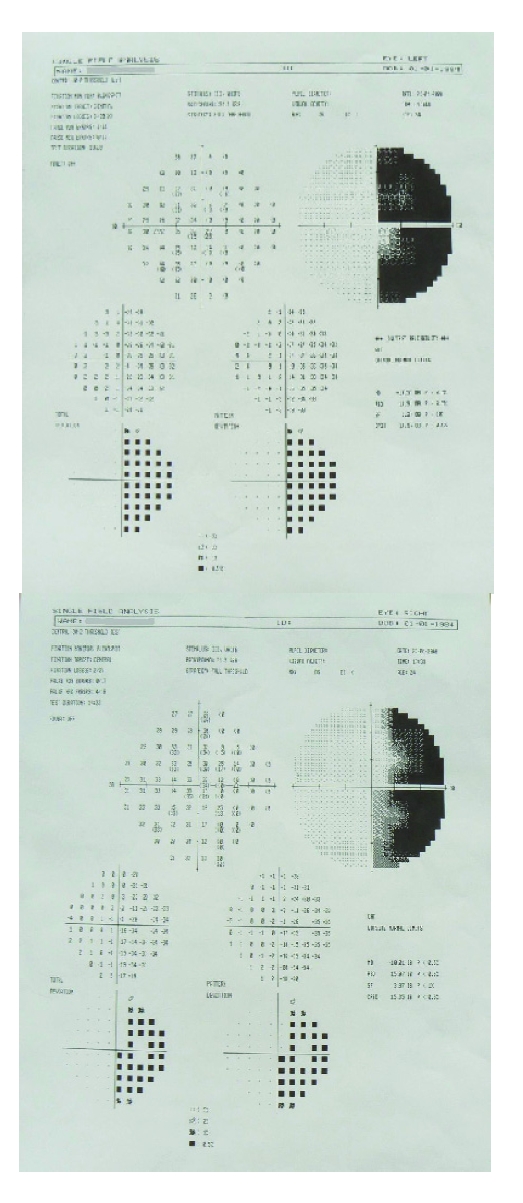
Visual field analysis shows left homonym hemianopia

**Figure 2:**
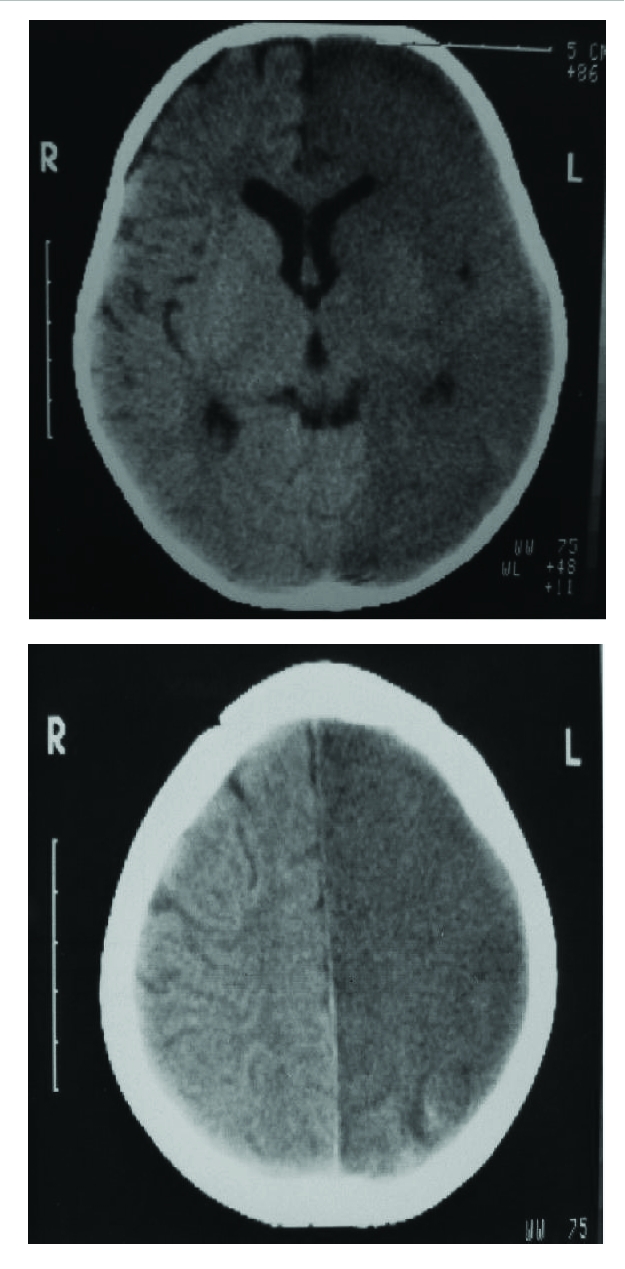
Initial Cranial CT shows total left– sided encephalomalasia

**Figure 3:**
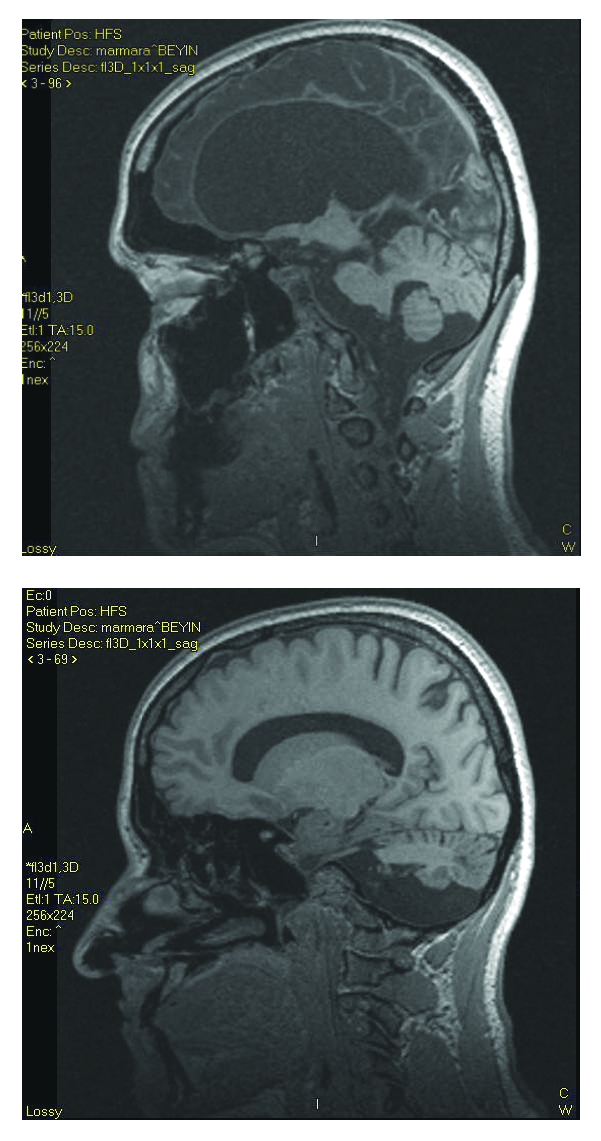
Cranial MRI shows total left hemisphere encephalomalasia which looked like the previous cranial CT

**Figure 4:**
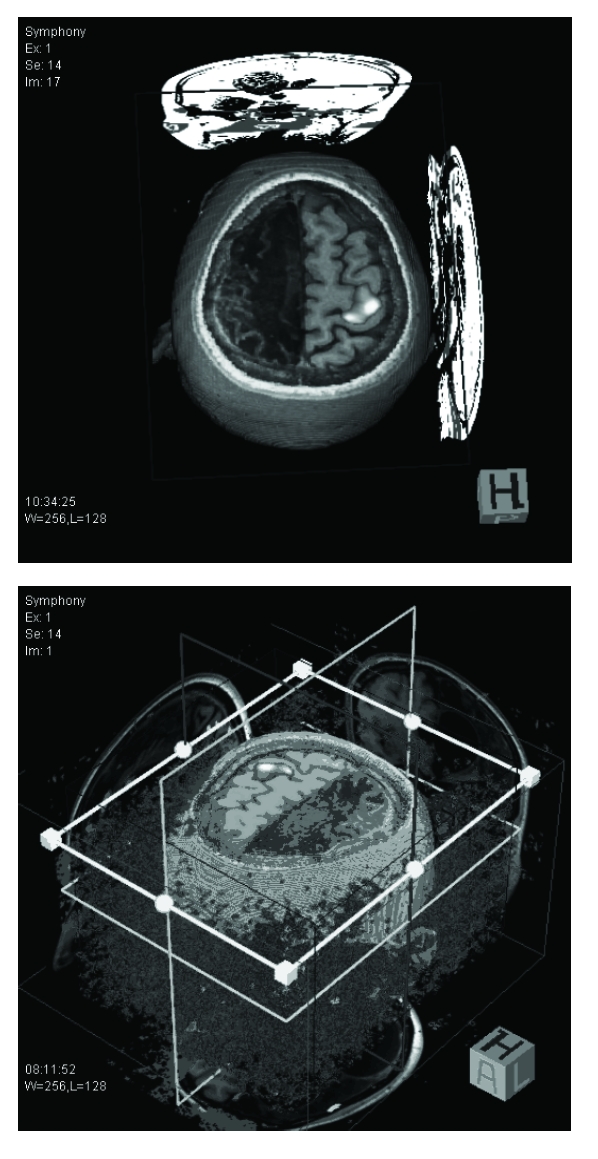
Functional MRI shows right hemisphere function on task–related brain activation during voluntary movement of patient's right leg

When she admitted to our hospital outpatient clinic, she was complaining of her right sided homonymous hemianopia and that it worsened in two days’ time. On the neurological examination she was speaking fluently, and cooperated on complicated comments. Motor examination revealed right–sided hemiparesis with increased tone predominantly on distal parts, right sided hyperreflexia and Babinski sign. At rest and particularly when she walked, her right foot inverted and assumed a dystonic posture. But she continued her activity of daily living without help. Her detailed neuropsychological examination revealed normal visual and spatial functions, mild impairment of executive functions (verbal and categorical fluency tasks) and broadening of simple attention (visual short and long–term memory and mental rotation) suggesting mild cognitive dysfunction. Cranial MRI showed total left hemisphere encephalomalasia which looked like the previous cranial CT (Figure 3). Blood and urine screening for inborn errors of metabolism, thyroid function studies, vasculitis panel were unremarkable. EEG showed left hemispheric slowing on the background without epileptiform discharge. As she was not capable of performing right hand function tasks by herself, functional MR imaging was performed with right leg function tasks. Right hemisphere function was noticed on task-related brain activation during voluntary movement of her right leg on functional MRI (Figure 4).

## Discussion

Extensive damage to the left hemisphere, which can be caused by stroke or tumor in adults, can result in profound and chronic aphasia. But when such a lesion occurred during childhood, especially before the complete maturation of brain, rarely results in pronounced speech and language disorders [6]. The mechanism under this event has been attributed to the plasticity and reorganization capacity of human brain which has been burdened by the many experimental and clinical studies. It is defined that developed brain reacts differently to the insults than adult brain [7]. 

Significant changes in the topography of the cortical somatosensory and motor maps have been demonstrated using non invasive mapping techniques as multichannel EEG, evoked potential, transcranial magnetic stimulation, functional magnetic resonance imaging and positron emission tomography [1]. Plasticity of motor representations have a major role in the recovery of motor function after stroke [8] and task related motor activation of unaffected motor cortex of the contralateral hemisphere in chronic stroke patients that has been reported by several previous studies [9]. However, the presence of ipsilateral projection from the undamaged hemisphere or a bilateral projection have been demonstrated with transcranial magnetic stimulation or functional MRI to the affected limb [10]. The biological age of the subject and the premorbid state of the brain influences the functional reorganization of the neuronal network [11].

Neuroimaging studies of both adults and children have indicated that right hemisphere language lateralization is more likely to be observed early rather than late. Liegeois and et al [6] was studied on children and adolescents epilepsy patients who sustained an early lesion in the left hemisphere, they also found that perilesional or remote reorganization for language other than right hemisphere function in functional MRI , but they concluded that non–left language lateralization is more common in patients who suffer from early or developmental left hemisphere lesions than in control subjects. 

Perilesional reorganization has also been observed in adults who suffered a left inferior frontal stroke and showed good language outcome [12]. The mechanism of functional recovery after CNS lesions has been tried to explain by the long term potentiation and depression, modulated by the up–down regulation of inhibitory and excitatory activity related to GABA, Ach and glutamate between other neurotransmitters [1]. Axonal and dendritic sprouting take place in animal models of lesional brain injuries, the neuronal plasticity in humans also may be related to this mechanism.

In the present case, there is an extensive damage to the left hemisphere but the clinical outcome is much better than expected. Functional MRI of the patient shows the adaptive changes of the human brain and permits the patients to live with mildly motor and cognitive dysfunctions. 

We could not find in the literature a lesion large enough to cause mild deficit although there is a total hemispheric damage.

## References

[1] Gomez–Fernandez L (2000). Cortical plasticity and restoration of neurologic function: an update on this topic. Rev Neurol.

[2] Staudt M, Grodd W (2002). Two types of ipsilateral reorganization in congenital hemiparesis: a TMS and fMRI study. Brain.

[3] Ward NS (2004). Functional reorganization of the cerebral motor system after stroke. Curr Opin Neurol.

[4] Ward NS (2005). Neural plasticity and recovery of function. Prog Brain Res..

[5] Ward NS (2005). Plasticity and the functional reorganization of the human brain. Int J Psyhophysiol.

[6] Liegeois F, Connelly A (2004). Language reorganization in children with early–onset lesion of the left hemisphere: an fMRI study. Brain.

[7] Lidzba K (2006). Visuospatial deficit in patients with early left–hemispheric lesions and functional reorganization of language: Consequences of lesion or reorganization?. Neuropsychologia.

[8] Butefisch CM (2004). Plasticity in the human cerebral cortex: lesions from the normal brain and from stroke. Neuroscientist.

[9] Calautti C (2003). Functional neuroimaging studies of motor recovery after stroke in adults: a review. Stroke.

[10] Thickbroom GW (2001). Differences in sensory and motor cortical organization following brain injury early in life. Ann Neurol.

[11] Fridman EA (2004). Reorganization of the human ipsilateral premotor cortex after stroke. Brain.

[12] Rosen HJ (2000). Neural correlates of recovery from aphasia after damage to left inferior frontal cortex. Neurology.

